# CopywriteR: DNA copy number detection from off-target sequence data

**DOI:** 10.1186/s13059-015-0617-1

**Published:** 2015-02-27

**Authors:** Thomas Kuilman, Arno Velds, Kristel Kemper, Marco Ranzani, Lorenzo Bombardelli, Marlous Hoogstraat, Ekaterina Nevedomskaya, Guotai Xu, Julian de Ruiter, Martijn P Lolkema, Bauke Ylstra, Jos Jonkers, Sven Rottenberg, Lodewyk F Wessels, David J Adams, Daniel S Peeper, Oscar Krijgsman

**Affiliations:** Division of Molecular Oncology, Netherlands Cancer Institute, Plesmanlaan 121, 1066 CX Amsterdam, The Netherlands; Central Genomic Facility, Netherlands Cancer Institute, Amsterdam, The Netherlands; Experimental Cancer Genetics, Wellcome Trust Sanger Institute, Hinxton, UK; Division of Molecular Genetics, Netherlands Cancer Institute, Amsterdam, The Netherlands; Division of Molecular Carcinogenesis, Netherlands Cancer Institute, Amsterdam, The Netherlands; Division of Molecular Pathology, Netherlands Cancer Institute, Amsterdam, The Netherlands; Center for Personalized Cancer Treatment, Amsterdam, The Netherlands; Department of Pathology, VU University Medical Center, Amsterdam, The Netherlands; Vetsuisse Faculty, Institute of Animal Pathology, University of Bern, Bern, Switzerland

## Abstract

Current methods for detection of copy number variants (CNV) and aberrations (CNA) from targeted sequencing data are based on the depth of coverage of captured exons. Accurate CNA determination is complicated by uneven genomic distribution and non-uniform capture efficiency of targeted exons. Here we present CopywriteR, which eludes these problems by exploiting ‘off-target’ sequence reads. CopywriteR allows for extracting uniformly distributed copy number information, can be used without reference, and can be applied to sequencing data obtained from various techniques including chromatin immunoprecipitation and target enrichment on small gene panels. CopywriteR outperforms existing methods and constitutes a widely applicable alternative to available tools.

**Electronic supplementary material:**

The online version of this article (doi:10.1186/s13059-015-0617-1) contains supplementary material, which is available to authorized users.

## Background

Genetic and epigenetic aberrations underlie many diseases and disorders. Recent advances in DNA sequencing technologies have facilitated the discovery of these changes, allowing disease gene discovery at an unprecedented rate [1,2]. Until now, the vast majority of efforts to uncover disease-genotype relations have deployed whole-exome sequencing (WES) or targeted sequencing on a smaller gene panel. These approaches enrich for the protein-coding sequences of the genome, or a subset of that, to focus the sequencing effort and reduce cost and data complexity compared to whole-genome sequencing (WGS) [3,4].

While WES has been successful in identifying disease-related mutations, it is well established that changes in copy number contribute to pathogenesis as well [5,6]. For this reason targeted sequencing efforts are commonly complemented with arrayCGH, SNP arrays, or low-coverage whole-genome sequencing (LC-WGS) [7-10], which increases the cost significantly. More recently, approaches that use reads mapping to captured regions (on-target reads) have been used as a cost-effective alternative to identify copy number aberrations (CNAs) [11-14]. Generally, the tools that apply this approach use the depth of coverage (DOC) of the captured exons as a measure of copy number status. Although DOC-based methods have proven useful for CNA detection from WGS data, their application to WES data suffers from two intrinsic limitations. First, copy number information from non-exonic regions is absent and can only be inferred from the exonic DOC. This impairs the discovery of CNAs in non-exonic regions, which have been shown to be clinically relevant [15-17]. Second, there is a large variation in the efficiency of capture baits to retrieve targeted sequences. All currently existing tools for DNA copy number profiling from WES apply sophisticated statistical models, such as principal component analysis, hidden Markov models, and singular value decomposition, for the segmentation and calling of genomic aberrations to overcome these limitations [11,12,18-21]. Despite this, the accuracy of copy number detection from WES is known to be poor when compared to methods that are dedicated to CNA detection [21]. These effects are even more pronounced when performing targeted sequencing with a small-size gene panel. Depending on the number of enriched genes, DNA copy number profiles will only yield sparsely distributed copy number information, which makes accurate downstream segmentation analysis more error-prone.

The enrichment strategies used for targeted sequencing generally achieve only 40% to 60% efficiency [22]; as a result, a large proportion of the sequence reads maps outside of these targeted regions. We hypothesized that such ‘off-target’ reads can be used to obtain DNA copy number profiles. This would provide not only more information on gene-poor regions, but also circumvent the aforementioned limitations of exonic DOC-based approaches.

Here, we describe the development of a new tool, called CopywriteR, which can be used to generate high quality DNA copy number profiles using off-target reads from targeted sequencing data. We analyze the performance of CopywriteR relative to other approaches based on on-target reads, and describe the wide applicability of the tool.

## Results

### Detection and removal of genomic regions enriched for sequence reads

**Figure 1:**
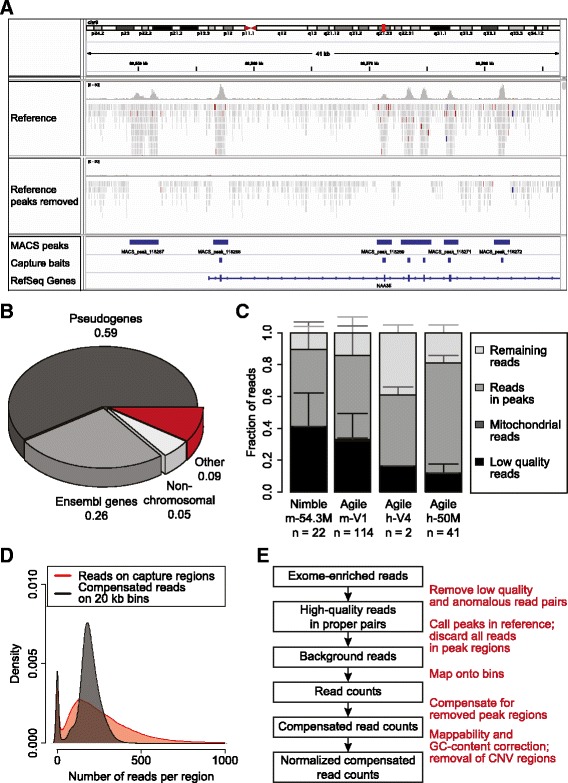
**Copy number information can be obtained from off-target reads. (A)** Screenshot from the IGV genome browser, showing an example of a genomic region with sequence reads mapping to the genome before and after removal of reads in Model-based Analysis for ChIPseq (MACS) called peaks. In addition, the location of MACS-peaks, capture regions, and genes are shown. **(B)** Germline DNA sample C41 was subjected to WES with capture set Agilent SureSelect Human Exon Kit V4. The nature of MACS-peaks that do not overlap capture regions is displayed. The fraction of these orphan peaks that overlap with pseudogene or Ensembl exons, that do not map to any of the reference genome chromosomes, that are unmappable, and that do not belong to any of these categories are shown. **(C)** The distribution of sequence reads of both germline and tumor DNA samples is shown for the indicated capture sets. Sequence reads are classified into one of these categories: (1) low mapping quality reads (Phred-score < 37 and/or reads do not pair properly); (2) mitochondrial reads; (3) reads in MACS-peaks; (4) remaining reads. Error bars represent standard deviations. **(D)** Germline DNA sample C45 was subjected to WES, and the amount of reads after compensation for reduced effective bin size is calculated and compared to the corresponding read counts from an exon-based method. Density plots of the number of sequence reads per data point are shown for each method. **(E)** A flowchart of the steps incorporated in the CopywriteR tool.

In an attempt to determine whether off-target reads from targeted sequencing can be used to derive copy number profiles, we first analyzed whole-exome sequencing (WES) data of a germline DNA sample (C41; for information about the datasets used in this manuscript, refer to Additional file 1). We discarded reads mapping to genomic regions covered by capture baits and sequences adjacent to these capture regions, which are frequently co-enriched during the capture procedure. We then calculated the number of reads that map to genome-wide consecutive 20 kb windows (bins) to obtain the depth of coverage (DOC). As the effective bin size was reduced upon removal of reads mapping to capture regions, we calculated a compensated DOC by dividing the DOC by the ratio of the effective bin size (that is, bin size minus the size of peak regions) to the original bin size (see Materials and methods). We removed sequences mapping within bait regions that were extended with up to 400 bp of flanking sequences. Despite this stringent filtering of the data, we were unable to eliminate capture biases and to generate smoothed copy number profiles (top four profiles in Additional file 2: Figure S1). We observed that a number of peaks of sequence reads on the genome were not overlapping with capture bait regions (for an example, see Figure 1A), which could explain the failure to generate high quality copy number profiles using this method.

Therefore, we decided to use off-target reads for copy number aberration (CNA) detection and applied the Model-based Analysis for ChIPseq (MACS) algorithm to detect peaks in germline samples enriched using various capture sets (see Additional file 1). First, we calculated that 125,000 to 170,000 MACS-peaks overlap with capture regions, depending on the capture set used. While the majority of exons targeted by baits were found within these MACS-peaks, we observed that 8% to 11% of peaks were located in non-exonic regions (Additional file 2: Figure S2A). Whereas the amount of peaks called by MACS was dependent on the number of sequence reads, a maximum was reached for samples with a total of 100 million reads. This suggests that the number of ‘orphan’ peaks is limited and that they represent parts of the genome that are reproducibly captured during target enrichment. We speculated that a fraction of orphan peaks was caused by co-capture of homologous sequences during exome enrichment. Therefore, we calculated the overlap of MACS-peaks with exons of both pseudogenes [23] and Ensembl genes [24]. Indeed, about 60% and 25% of orphan peaks constituted pseudogenes or untargeted Ensembl genes, respectively (Figure 1B). This was more than what would be observed by chance, as 10,000 simulations using randomly placed peaks of the same size distribution did not once reach the observed extent of overlap with either pseudogene or Ensembl exons (Additional file 2: Figure S2B and C). We observed a similar effect for other capture sets (Additional file 2: Figure S2D and E), which suggests that the majority of orphan peaks can be explained by sequence co-capture during exome enrichment strategies. As such orphan peaks could introduce noise into the copy number data, we decided to filter sequence reads in MACS-peaks (termed ‘peak removal’ in the remainder of the manuscript). This indeed led to a dramatic noise reduction (compare top four profiles to bottom profile in Additional file 2: Figure S1). Global inspection using the IGV genome browser showed that this procedure efficiently removes distinguishable peaks (Figure 1A).

### Off-target sequence reads are uniformly distributed over the genome

Next, we tested whether sufficient sequence reads were left for CNA calling after peak removal. We noticed that among the different capture sets, at least 10% of the input sequence reads were reads with high mapping quality (Phred-score >37) outside of MACS-peak regions (Figure 1C). From our experience with low-coverage whole-genome sequencing (LC-WGS) approaches with 20 kb bins, 5 million reads are sufficient for accurate CNA detection. This is in line with the number of reads that map outside of MACS-peak regions (Additional file 2: Figure S3). We found that 86.6% of our samples had more than 5 million useable off-target reads, suggesting that most exome sequence datasets will be amenable to analysis using our approach.

We subsequently tested whether the off-target reads display a uniform distribution over the genome in a set of six human germline DNA samples. For this, we performed MACS-based peak calling with subsequent peak removal followed by the application of the 20 kb bin DOC-based approach with a compensated DOC (see above and Materials and methods). Although in theory this compensation could lead to a bias, there was no obvious relationship between the compensated DOC and the effective bin size in male sample C45 (Additional file 2: Figure S4; upper panel). The non-random scattering pattern of the effective bin sizes was due to clustering of bins where the same number of MACS-peak regions has been removed (Additional file 2: Figure S4; lower panel). When comparing the compensated off-target DOC to the DOC on capture regions (black and red, respectively; Figure 1D and Additional file 2: Figure S4), the density distribution of the off-target DOC appeared normally distributed and was relatively narrow.

In addition to the above, a small shoulder could be distinguished representing the sex chromosomes (Figure 1D and Additional file 2: Figure S4). Therefore, based on the notion that we can distinguish different copy number states from off-target reads, we developed ‘CopywriteR’ (Figure 1E), which exploits off-target reads from targeted sequencing for CNA detection. This method is based on peak calling using MACS in a matched reference sample or, when no reference is available, in the sample itself. Sequence reads in peaks are removed, and the DOC is calculated based on fixed-size bins. The DOC is compensated for peak removal, normalized using loess-based corrections for mappability and GC content, and filtered for regions of extensive germline copy number variation (see Materials and methods). CopywriteR is implemented in R and is available for download from GitHub [25].

### Comparison of CopywriteR and methods dedicated to copy number detection

**Figure 2:**
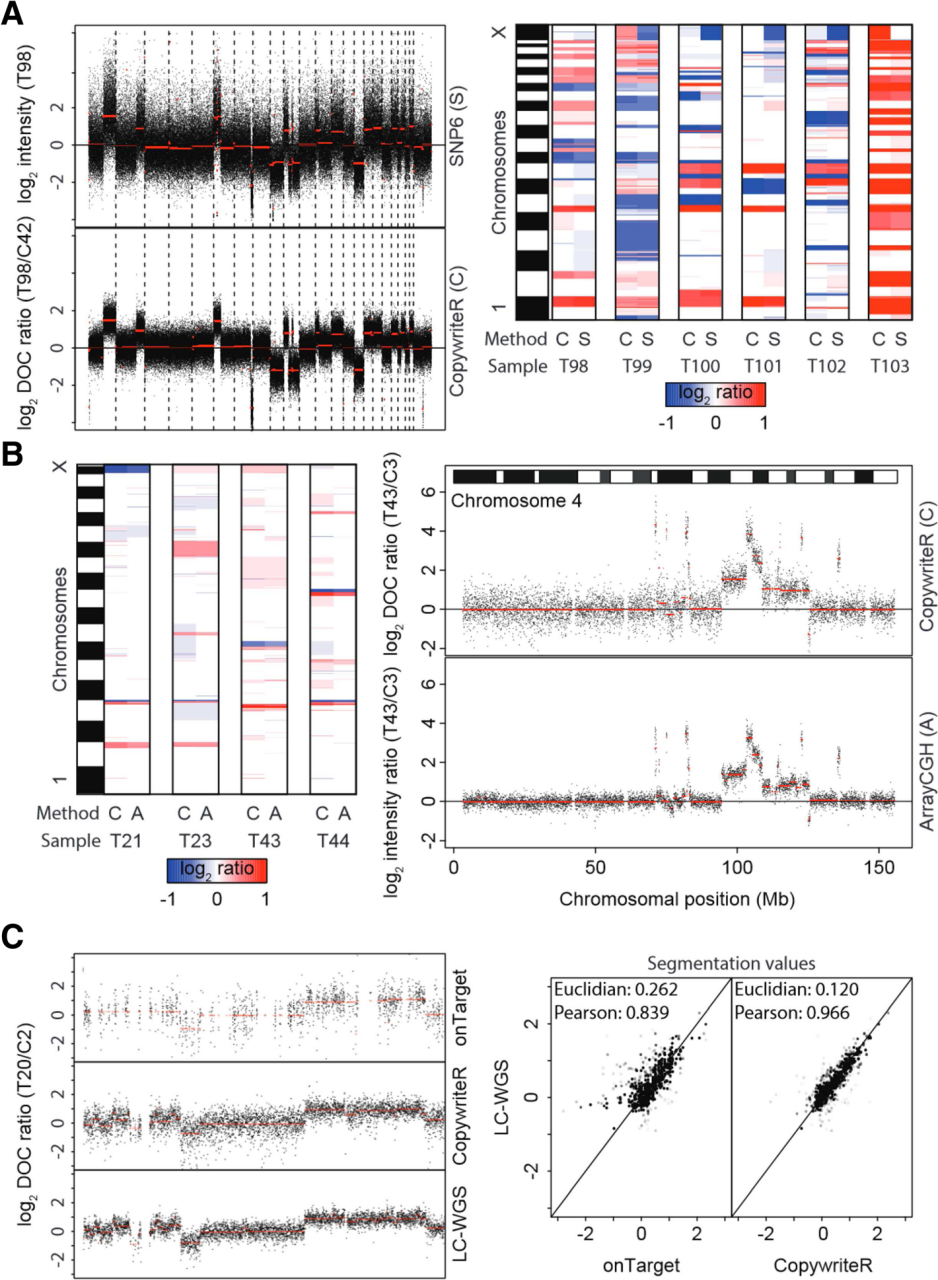
**CopywriteR compares to dedicated copy number detection methods. (A)** Six PDX-derived human melanoma were subjected to WES and analyzed on SNP6 arrays. Pseudo counts were derived (see Materials and methods), and used as a basis for copy number profiles, with segmentation values (CBS) depicted in red (left panel). After segmentation, segmentation values were represented as a heatmap to show concordance of the two methods. **(B)** Four murine small-cell lung carcinomas (SCLC) were subjected to WES and analyzed by arrayCGH. Pseudo counts were created and used for creating copy number profiles, with segmentation values (CBS) depicted in red (right panel). Segmentation values were plotted as in **(A)** for comparison of the two methods (left panel). **(C)** Tumor T20 from a breast cancer mouse model was subjected to WES or LC-WGS. Copy number profiles of chromosome 12 generated with onTarget or CopywriteR methods are compared to the profile from LC-WGS data of the same material, with segmentation values (CBS) depicted in red (left panel). Segmentation values of onTarget and CopywriteR methods are plotted against the LC-WGS method, and Euclidian distances and Pearson correlation coefficients of segmentation values are displayed (right panel).

Next, we assessed the performance of CopywriteR relative to dedicated copy number detection platforms. Six PDX-derived human melanoma samples (T98-T103) were analyzed with Affymetrix SNP6 chips and WES using the same DNA preparation. To allow a direct comparison of the copy number data from CopywriteR and Affymetrix SNP6, we generated pseudo counts of the SNP6 data as described previously [11-14,20] and in the Materials and methods. As an example, the copy number profile of sample T98 was very similar for both methods (Figure 2A; left panel). In a side-by-side comparison, the segmentation values for the two methods were nearly identical for all samples (Figure 2A; right panel). However, the variance of the SNP6 profiles was higher (Figure 2A; left panel), as was reflected by the MAD values for SNP6 (mean = 0.56, range = 0.46 to 0.73) and CopywriteR (mean = 0.26, range = 0.24 to 0.27). Since the deflection (that is, the difference between two copy number states) of aberrations between CopywriteR and SNP6 was similar, the sensitivity of detection of CNAs was higher for CopywriteR. This was also reflected by the higher signal-to-noise ratios for CopywriteR-derived copy number profiles compared to those of SNP6 (Table 1; [26]). In sum, while CopywriteR and SNP6 copy number profiles are largely comparable, the sensitivity for detecting gains and losses is higher when CopywriteR is used.

**Table 1:** **MAD values, signal, and signal-to-noise ratios of CopywriteR and SNP6-derived copy number profiles from the PDX-derived human melanoma sample set**

	**CopywriteR**			**SNP6**			
	**MAD**	**Signal**	**SNR**	**MAD**	**Signal**	**SNR**	**Genomic location**
T98	0.206	0.722	3.50	0.547	0.721	1.32	chr6p
T99	0.241	0.344	1.43	0.610	0.302	0.50	chr16
T100	0.225	0.618	2.75	0.680	0.649	0.95	chr1q
T101	0.252	0.996	3.95	0.927	0.829	0.89	chr1q
T102	0.248	0.592	2.39	0.753	0.617	0.82	chr8q
T103	0.237	0.978	4.13	0.516	0.931	1.80	chr2q

To compare the quality of CopywriteR-derived data to those from arrayCGH, we analyzed DNA of four mouse small cell lung cancer (SCLC) samples (T21, T23, T43, and T44) with both Nimblegen 135 K arrays and WES using the same DNA preparation. For a direct comparison of copy number profiles of CopywriteR and Nimblegen array methods, we matched the array probes to the nearest bin center to create pseudo counts. Copy number profiles were plotted together with their segmentation values for both methods (Figure 2B). On the whole-genome level, all aberrations were detected in arrayCGH and CopywriteR-derived data (Figure 2B; left panel). We observed that chromosome four of sample T43 displays a complex copy number profile with multiple high-level amplifications (Figure 2B; right panel). Comparison between the techniques showed that copy number profiles derived from the Nimblegen array platform and from CopywriteR were highly similar, with all major aberrations detected with both methods. The signal-to-noise ratio was slightly higher in the arrayCGH datasets (Table 2). While the technical noise was lower for the array (mean MAD = 0.24, range = 0.22 to 0.26) compared to CopywriteR (mean MAD = 0.49, range = 0.42 to 0.61), the biological signal, as denoted by the deflection, was higher for CopywriteR. The lower deflection of copy number profiles obtained from Nimblegen arrays in comparison with other DNA copy number platforms has been described before [27,28]. In sum, CopywriteR accurately detects CNAs and performs similar to the dedicated DNA copy number technique arrayCGH.

**Table 2:** **MAD values, signal, and signal-to-noise ratios of CopywriteR and arrayCGH-derived copy number profiles from the murine small-cell lung cancer sample set**

	**CopywriteR**			**Nimblegen**			
	**MAD**	**Signal**	**SNR**	**MAD**	**Signal**	**SNR**	**Genomic location**
T21	0.351	0.585	1.67	0.246	0.49	1.99	chr2: 141-182 Mb
T22	0.341	0.532	1.56	0.276	0.493	1.79	chr2: 147-182 Mb
T23	0.479	-0.626	1.31	0.219	-0.443	2.02	chr7: 116-153 Mb
T24	0.580	1.115	1.92	0.274	0.737	2.69	chr10: 80-108 Mb

To further assess the data quality obtained using CopywriteR, we tested our approach on a set of nine mouse mammary tumors for which both WES (Nimblegen or Agilent capture sets) and LC-WGS data were available. We applied CopywriteR to the WES set, and compared the profiles with those obtained from LC-WGS (Figure 2C). CopywriteR-based profiles were highly similar to those obtained from LC-WGS data, and while the technical noise was higher for CopywriteR compared to LC-WGS (MAD values of 0.50 versus 0.32), a part of this might be explained by the lower read count for CopywriteR-derived copy number profiles. Across all samples, the MAD values were marginally higher for the CopywriteR method (mean MAD = 0.38, range = 0.28 to 0.67) compared to LC-WGS (mean MAD = 0.27, range = 0.19 to 0.60), which is reflected by the lower signal-to-noise ratio for LC-WGS (Table 3).

**Table 3:** **MAD values, signal, and signal-to-noise ratios of CopywriteR, onTarget, and LCWGS-derived copy number profiles from the murine breast cancer sample set**

	**CopywriteR**			**LC-WGS**			**onTarget**			
	**MAD**	**Signal**	**SNR**	**MAD**	**Signal**	**SNR**	**MAD**	**Signal**	**SNR**	**Genomic location**
T60	0.262	0.523	2.00	0.176	0.501	2.85	0.159	0.467	2.94	chr6: 52-120 Mb
T56	0.250	0.494	1.98	0.170	0.513	3.02	0.172	0.568	3.30	chr16: 12-25 Mb
T20	0.314	1.099	3.50	0.271	0.978	3.61	0.653	1.135	1.74	chr8:7-16 Mb
T62	0.265	0.421	1.59	0.185	0.403	2.18	0.160	0.376	2.35	chr8: 3-47 Mb
T19	0.252	1.314	5.21	0.163	1.272	7.80	0.591	1.24	2.10	chr8:22-39 Mb
T2	0.395	0.303	0.77	0.255	0.335	1.31	0.230	0.344	1.50	chr5:38-152 Mb
T3	0.293	0.401	1.37	0.212	0.361	1.70	0.181	0.286	1.58	chr15:87-104 Mb
T7	0.597	0.395	0.66	0.538	0.398	0.74	0.330	0.371	1.12	chr6
T50	0.262	0.477	1.82	0.193	0.486	2.52	0.175	0.462	2.64	chr5

To investigate how CopywriteR performs relative to other methods that are based on on-target reads, we implemented our own version of the exonic DOC-based method ExomeCNV [12], which processes copy number data according to the CopywriteR workflow where possible (we refer to this method as ‘onTarget’ in the remainder of the text; see Materials and methods for details). For sample T20, we observed that segmentation of onTarget data did not allow detection of all aberrations, as was most clearly shown for parts of the p-arm of chromosome 12 (Figure 2C; left panel). In addition, when comparing to the onTarget method, the segmentation values of CopywriteR correlated better with LC-WGS, as was reflected by a higher Pearson correlation coefficient and a lower Euclidian distance. Thus, from this analysis it appears that CopywriteR outperforms the onTarget method.

### CopywriteR outperforms exonic read-based methods for copy number detection

To extend our analysis of the potential of CopywriteR, we next analyzed the entire sequence dataset of nine mouse mammary tumors, and measured the performance of CopywriteR and onTarget approaches based on downstream segmentation analysis. We included two of the most recent and best segmentation algorithms, propSeg [20,21] and circular binary segmentation (CBS) [29]. In addition, we included EXCAVATOR, a tool dedicated to obtaining copy number information from WES efforts. For this analysis, we compared the performance to results obtained from LC-WGS data.

**Figure 3:**
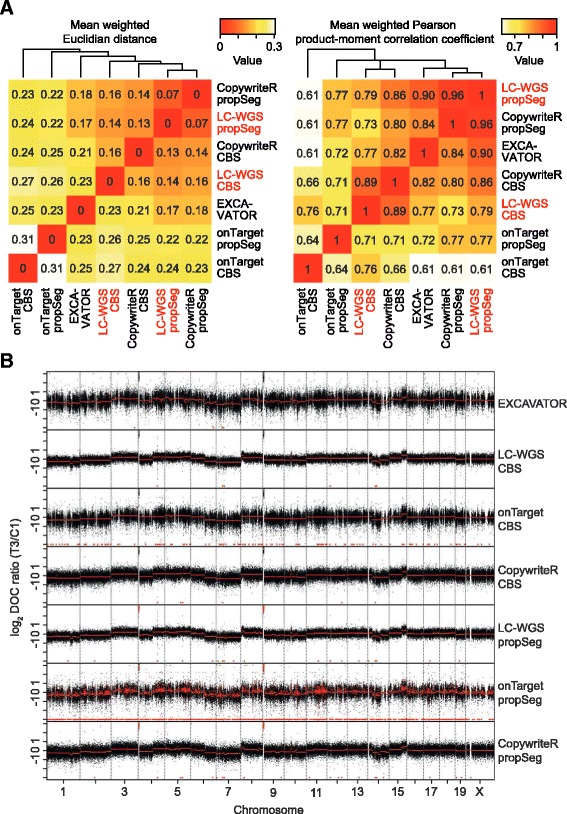
**CopywriteR outperforms exonic depth of coverage-based methods. (A)** Tumors from a breast cancer mouse model were subjected to WES or LC-WGS, and analyzed using CopywriteR or onTarget methods. Subsequently, copy number data were segmented using propSeg or CBS, while the integrated EXCAVATOR tool was used in addition. Weighted Euclidian distances (left) and Pearson correlation coefficients (right) were calculated between the different approaches for every sample, and the means of those values across all samples are represented as clustered heatmaps. **(B)** As in **(A)**; the genome-wide copy number plots for sample T3 are displayed for the indicated analysis methods, with segmentation values depicted in red.

To assess the similarity between these methods and the LC-WGS reference, we derived the mean weighted variant of both the Euclidian distance and the Pearson correlation coefficient between each set of methods based on segmentation values obtained using CBS or propSeg. These measures were then used for clustering analysis (Figure 3A). We observed that LC-WGS and CopywriteR-derived copy number states were least distant, irrespective of the segmentation algorithm that was applied. Similarly, the correlation between LC-WGS and CopywriteR was highest, albeit that these approaches formed sub-clusters based on the applied segmentation approach. Also, when comparing copy number profiles and the resulting segmentation values, CopywriteR and LC-WGS data appeared most alike (Figure 3B). Thus, despite the variation in WES and LC-WGS reads among the samples (see Additional file 1), CopywriteR outperforms the onTarget approach, and the segmentation values of CopywriteR-derived copy number profiles are more similar to those of LC-WGS than other exonic read-based methods.

A possible limitation of CopywriteR may be that its performance depends on the effectiveness of target enrichment, as a higher capture efficiency would lead to fewer off-target reads, and therefore result in less accurate copy number information. To exclude this possibility, we obtained a set of 16 sequence files from The Cancer Genome Atlas (TCGA) with matching copy number information from SNP6 arrays (see Materials and methods), and performed a similar analysis. For the entire dataset, the mean weighted Euclidian distance of CBS-derived segmentation values of SNP6 was closest to those of CopywriteR. In addition, the mean weighted Pearson correlation coefficient SNP6 also correlated best with CopywriteR (Additional file 2: Figure S5A). Moreover, while the onTarget method suffered from ‘noisy’ segmentation, this was much less the case for SNP6 and CopywriteR approaches (Additional file 2: Figure S5B). Thus, together with our previous analyses, these results show that CopywriteR outperforms other exonic read-based approaches.

While comparing the onTarget and CopywriteR approaches on a dataset of six melanoma PDX (T98-T103) with matched germline samples, we observed two phenomena that could explain the better performance of CopywriteR. While the dispersion of the germline samples in the dataset (C43 to C47) was slightly lower for the onTarget method compared to CopywriteR (Additional file 2: Figure S6A; left panel), there were more outliers (data points >1.5× the interquartile range away from the first and third quartiles) with onTarget (Additional file 2: Figure S6A; right panel). These outliers appeared to specifically concentrate at discrete values, suggesting that they corresponded to capture regions with low DOC. Indeed, when we highlighted bins according to their absolute normalized compensated DOC in the tumor sample from low (red) to high (dark gray), we observed that the large majority of outliers obtained with the onTarget approach are regions with low DOC, while this was not the case for CopywriteR (Additional file 2: Figure S6B). Thus, whereas onTarget copy number profiles display differences in technical noise depending on the bait efficiency, CopywriteR does not suffer from this limitation.

Another explanation for CopywriteR’s better performance is that segmentation tools appear to perform better on CopywriteR-derived DOC. One problem with segmentation algorithms is the sheer number of segments that are called, with over-segmentation frequently occurring. The CBS algorithm appointed a limited number of segments on CopywriteR-based data for each of the tumor samples (T98 to T103), and the number was comparable to that obtained using EXCAVATOR. In contrast, application of the onTarget method resulted in clear over-segmentation (Additional file 2: Figure S7A and B). Thus, in contrast to CopywriteR-derived copy number information, on-target DOC constitutes a poor substrate for traditional segmentation algorithms. In sum, CopywriteR outperforms other exonic read-based methods, potentially due to both the presence of a high number of outliers in on-target data and the poor performance of traditional segmentation algorithms on these types of data.

### CopywriteR allows for copy number detection without a reference

**Figure 4:**
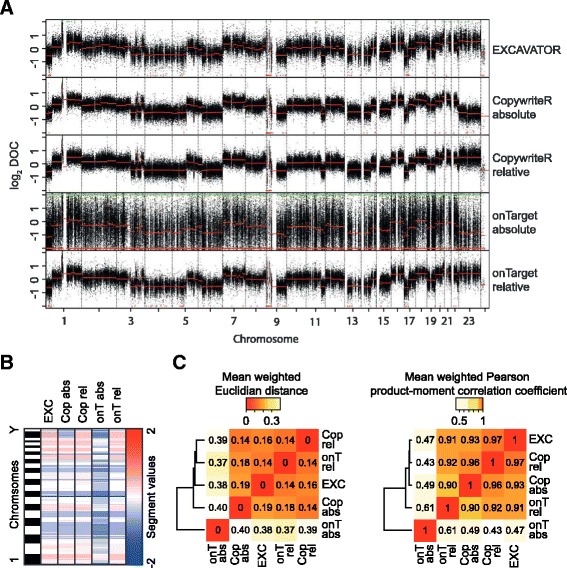
**Copy number detection in the absence of a reference. (A)** CopywriteR and onTarget methods were applied to WES data of melanoma PDX sample T99, either with or without C43 as a reference. Genome-wide copy number profiles are shown, with segmentation values (CBS) depicted in red. **(B)** CBS-derived segmentation values of the analysis in **(A)** are represented in a heatmap. **(C)** Segmentation values of all six melanoma PDX samples were treated as in **(A)** and **(B)**, and the weighted Euclidian distances and Pearson correlation coefficients were calculated for every sample between the different methods. The means of those values across all samples are represented as clustered heatmaps.

Patient samples are frequently subjected to WES, together with a matched germline reference sample for variant detection. In some cases, however, a reference sample is absent. A similar limitation applies to cultured cell lines and archival tissue, for which reference material is rarely available. Others have already shown that LC-WGS can be used to generate copy number profiles without the need for a reference [10,22,30,31]. CopywriteR mimics LC-WGS in that it uses uniformly distributed sequence reads. Therefore, we tested the feasibility of using CopywriteR without a reference. We analyzed melanoma PDX sample T99 using CopywriteR and onTarget methods, both with and without a matching reference (‘relative’ and ‘absolute’, respectively). Segmentation was subsequently performed using CBS. EXCAVATOR does not have an implementation to perform the analysis without a reference sample and was therefore only tested with a reference. All five methods were able to capture the main somatic copy number aberrations (Figure 4A). As expected, the onTarget-absolute method was highly noisy due to the differences in bait efficiency. In contrast, application of CopywriteR-absolute resulted in copy number profiles that were highly identical to any of the relative profiles, and this was also the case for the segmentation values obtained using CBS (Figure 4B). We then analyzed the segmentation values for the entire melanoma PDX dataset in terms of the Euclidian distance and the Pearsons correlation (Figure 4C). While the onTarget-absolute method displayed limited similarity to the approaches using a reference, CopywriteR-absolute was highly concordant with the relative methods. Thus, these analyses show that CopywriteR is unique in its ability to extract accurate copy number information from targeted sequencing data without a reference.

### CopywriteR can be applied to sequencing data from various sources

**Figure 5:**
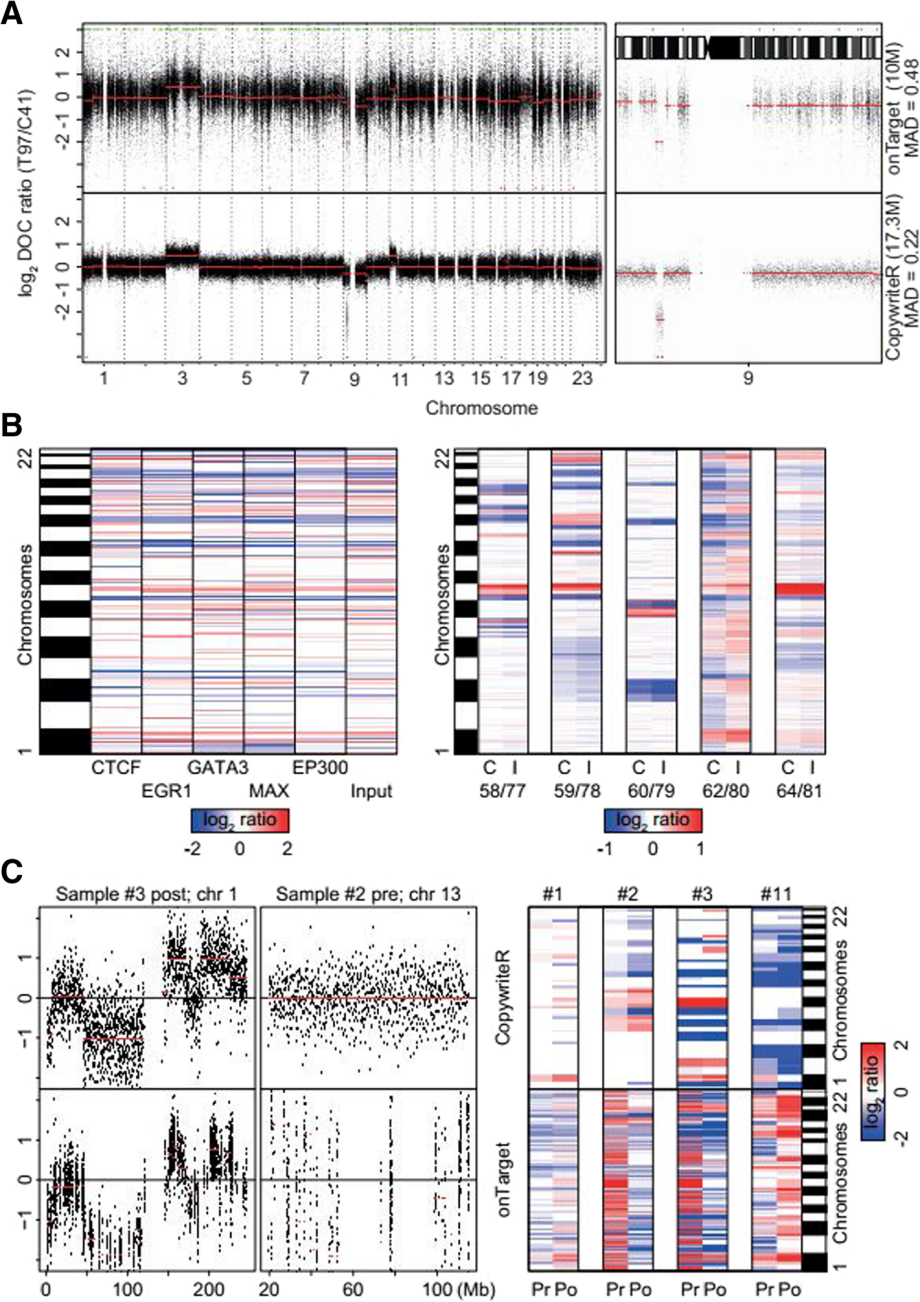
**CopywriteR is widely applicable. (A)** Sample T97 (FFPE) was subjected to WES, and copy number profiles relative to C41 (fresh frozen reference material) are displayed for onTarget and CopywriteR methods, with segmentation values (CBS) depicted in red (left panel: whole-genome; right panel: chromosome 9). **(B, left panel)** ChIPseq data were obtained from ChIP experiments on the MCF7 cell line with the indicated set of antibodies, or from the relevant input control. Copy number data were extracted using CopywriteR, and further analyzed employing CBS. Segmentation values are represented as a heatmap. **(B, right panel)** Data were analyzed as for the left panel. ChIPseq data were obtained from ChIP experiments on ER^+^ breast cancer with ER-antibodies (E), or from the relevant input (I) control. **(C, left panel)** A set of matched pre- and post-vemurafenib treatment melanoma samples were subjected to targeted sequencing on a 1,977-gene panel. Copy number information was extracted using CopywriteR and example regions of the resulting copy number profiles are presented, with segmentation values (CBS) depicted in red. **(C, right panel)** Segmentation values were plotted as a heatmap for the pre/post-treatment pairs.

All samples that we analyzed thus far were from frozen material and thus based on high-quality DNA. Since (tumor) tissue is often archived as formalin-fixed, paraffin-embedded (FFPE), we challenged CopywriteR’s performance with sequencing data obtained from DNA of such suboptimal quality. We compared the performances of CopywriteR and onTarget on WES data obtained from DNA of an FFPE melanoma biopsy. We observed that the technical noise in the profile of the onTarget method was higher compared to the CopywriteR method (Figure 5A), which was also reflected by higher MAD values for onTarget (0.52) relative to CopywriteR (0.22). In addition, the onTarget method displayed GC-content bias (compare the GC-rich chromosomes 19 and 22 with GC-poor chromosomes 13 and 18 [32,33]), which was absent from the profiles that were analyzed with CopywriteR. Thus, the better performance of CopywriteR is also observed for samples of sub-optimal DNA quality.

ChIPseq is a widely used technique combining chromatin immunoprecipitation (ChIP) with NGS to identify the binding sites of DNA-associated proteins. Because the enrichment of genomic regions containing such binding sites is relatively poor, we hypothesized that sufficient off-target reads would be available for CopywriteR to derive accurate copy number profiles. We tested this on the breast cancer cell line MCF7 for which sequence data from both input material as well as multiple ChIPseq experiments for a number of DNA-associated proteins (ER, EGR1, GATA3, CTCF, MAX, and EP300) are available [34]. Using downstream segmentation analysis with CBS, we observed that CopywriteR accurately and reproducibly detected copy number changes (Figure 5B; left panel and Additional file 2: Figure S8).

In addition, we analyzed five publically available ChIPseq samples [35] using CopywriteR and CBS, and compared the resulting copy number information to that from the input material. DNA copy number profiles of 20 kb resolution were very similar between CopywriteR and input material-derived data, as illustrated in a heatmap (Figure 5B; right panel) and on genome-wide copy number profiles (Additional file 2: Figure S9). In sum, CopywriteR not only performs well on WES data, but also on other types of next-generation sequencing data, such as those obtained from ChIPseq.

Based on the performance of CopywriteR on WES, we wanted to further challenge CopywriteR and test its performance on targeted sequencing of a small-size gene panel. We used a set of biopsies from four patients with BRAF^V600E^-mutant melanoma, where for each patient, samples before and after vemurafenib (a clinical BRAF inhibitor) treatment, as well as matching normal references, are available. Samples were sequenced following target enrichment for 1,977 genes (29,596 exons; 115,332 baits; [36,37]). While the percentage of off-target reads was similar to that of WES (3.0% to 12.0%), the total amount of reads (26.9 to 74.9 million reads) was lower in this dataset. As a result, the number of off-target reads was also relatively low; we therefore increased the bin size to which CopywriteR analysis was applied to 100 kb (Figure 5C; left panel and Additional file 2: Figure S8). For the onTarget method, copy number information was sparsely available, and data segmentation was inaccurate as evidenced from over-segmentation of the data (155 to 459 segments). In contrast, CopywriteR produced evenly distributed copy number information across the entire genome, with accurate segmentation as a result (26 to 53 segments). Although differences in copy number states might have occurred between the pre- and post-treatment samples, a heatmap of the segmentation values demonstrated that they were highly similar for CopywriteR but not onTarget-derived copy number data (Figure 5C; right panel). Thus, while the inherent focus of targeted sequencing for a small-size gene panel is limited to a few regions of the genome, CopywriteR is uniquely able to expand this focus and obtain accurate genome-wide copy number information.

## Discussion

Targeted sequencing is commonly used in cancer research and clinical genetics [8,38], mainly for mutation detection. Such sequencing data can also be used to extract DNA copy number information, and the demand for this has led to the recent development of numerous exonic DOC-based methods (for a review see [21]). However, the non-uniform genomic distribution of captured exons and the differences in bait efficiency limit the detection of CNAs in gene-poor regions, and constitute challenges for downstream analysis.

Here, we describe an alternative to the exonic DOC-based methods available for CNA detection from targeted sequencing data. By using the off-target reads that are discarded by conventional methods, we generated high quality and reproducible DNA copy number profiles. The data quality of CopywriteR-derived copy number information is similar to, or outperforms, dedicated copy number profiling platforms (Figure 2). In addition, CopywriteR performs better than exonic read-based methods with respect to the accuracy of segmentation (Figure 3 and Additional file 2: Figure S5). Thus, CopywriteR constitutes a better alternative to existing tools for obtaining copy number information from WES.

CopywriteR addresses two main problems of using exon-based DOC. First, CopywriteR is independent of the genomic position of capture baits. As opposed to exonic read-based approaches, CopywriteR will provide copy number information on any region in the genome, including for gene-poor regions. Second, the independence of CopywriteR from bait efficiency has the advantage that it permits analysis of samples without a reference. In sharp contrast, exon-based DOC methods require a reference, as differences in bait efficiency preclude the use of ‘single-channel’ copy number profiles. One could compensate for these differences using a correction based on earlier sequence data using the same capture set. However, it remains to be shown whether this is feasible, as bait efficiencies would need to be constant throughout independent experiments.

The applicability of CopywriteR extends beyond WES, as we have shown that CopywriteR can be applied to other data types as well, including ChIPseq and targeted sequencing of small-size gene panels. Preliminary data suggest that also FAIREseq data are a good substrate for CopywriteR analysis. Thus, our data suggest that CopywriteR can be used to extract copy number information from many other enrichment strategies too.

CopywriteR opens the possibility to extract accurate genome-wide DNA copy number profiles, also in settings where this could previously only be reached by means of additional experiments and expenses. For instance, we show that CopywriteR performs well on targeted sequencing of small-size gene panels, that it has the unique ability to perform well on FFPE archival tissue, and that it allows extraction of copy number information without the need for a reference. These features are unique to CopywriteR and are clinically highly relevant, since targeted sequencing is the sequencing type of frequent choice in diagnostic settings, where archival FFPE tissue often lacks reference material. Thus, CopywriteR has the potential to extract important additional information from both existing and new sequencing efforts, thereby unlocking the full potential of sequencing data.

## Conclusions

Here, we present a novel tool, called CopywriteR, for the detection of copy number aberrations from targeted sequencing. All currently available methods are based on exonic depth of coverage, and suffer from the problems that bait efficiencies are non-uniform and that exons are irregularly distributed over the genome. By exploiting the off-target sequence reads, CopywriteR bypasses these problems. It allows for extracting DNA copy number profiles of a high quality comparable to those of ‘dedicated’ techniques such as SNP array, arrayCGH, and low-coverage whole-genome sequencing techniques. CopywriteR outperforms exonic read-based approaches and has the ability to derive copy number information even in the absence of a reference. CopywriteR is widely applicable on sequence data ranging from ChIPseq to targeted sequencing on a small-size gene panel. Without the need for additional experiments and expenses, CopywriteR opens new possibilities to further mine both existing and future sequencing efforts.

## Materials and methods

### Sample selection

CopywriteR was evaluated against WES, targeted sequencing, and ChIPseq datasets. The WES datasets includes: (1) six PDX-derived human melanoma samples with six matched germline references (T98 to T103; C42 to C47); (2) four mouse SCLC samples with one matched germline reference (T21, T23, T43, and T44; C3); (3) nine mouse mammary tumors with two matched germline references (T2, T3, T7, T19, T20, T50, T56, T60, T62; C1, C39); (4) one melanoma biopsy (FFPE) with matched reference (T97; C41); and (5) 16 kidney renal cell carcinoma (KIRC) samples downloaded from the TCGA together with matching germline references [8]. The targeted sequencing dataset includes matching pre- and post-vermurafenib treatments melanoma samples, as well as germline references [37]. The ChIPseq datasets include: (1) the breast cancer cell line MCF7 which was analyzed with multiple ChIPseq samples with six DNA associated proteins (ER, EGR1, GATA3, CTCF, MAX, and EP300) as well as input material as a control [34]; and (2) five breast cancer samples enriched for ER-binding sites and matching input control samples [35]. Detailed information (including origin, sequencing method, sequence depth, and other sequencing statistics) for all samples is documented in Additional file 1.

In addition to sequencing data, PDX-derived melanoma and TCGA data were analyzed using Affymetrix SNP6; mSCLC samples were analyzed on Nimblegen arrays; mouse mammary tumors were analyzed by WG-LCS. The Animal Experimental Committee approved all animal experiments. Samples were collected following approval of the Medical Ethical Committee of the NKI (study code N03LAM) and in compliance with the Helsinki Declaration. Previously unpublished data have been made available through the NCBI Gene Expression Omnibus (GEO) [39] (accession number GSE60259), the European Nucleotide Archive (ENA) [40] (accession number PRJEB6954), and the European Genome-phenome Archive (EGA) [41] (accession number EGAS00001000617).

### Data processing

Segmentation of all copy number profiles was calculated using CBS [29] as implemented in the R-package CGHcall 2.22.0 [42], except where indicated using propSeg [20]. The median absolute deviation (MAD) was calculated using madDiff from the R-package matrixStats 0.10.0 [43]. Signal-to-noise ratios were calculated by dividing the absolute segmentation value for a large segmented genomic region (called using CBS) by its MAD value. All human and mouse data were mapped onto the hg19 and mm10 reference genomes, respectively. Random sampling of sequence reads was performed using SAMtools view -s 0.1.18 [44].

#### Whole-exome sequencing

DNA libraries were prepared using the Illumina Paired End Sample Prep Kit according to the manufacturer’s protocol. Target enrichment was performed using the Nimblegen SeqCap EZ Mouse 53.4 Mb, Agilent SureSelect Mouse all Exon Kit V1, Agilent SureSelect Human Exon Kit V4, and Agilent SureSelect Human Exon Kit 50 Mb capture sets. Sequencing was performed on Illumina HiSeq 2000 sequencers. Reads were mapped by bwa 0 to 7.5 [45] with default settings. SAM files were processed using Picard 1.101 [46], SAMtools and the Genome Analysis ToolKit (GATK) release 2.7-4 [47]. In brief, SAM files were binary compressed, sorted, and indexed by SAMtools (samtools view, sort, and index tools), duplicated reads were removed by Picard (with MarkDuplicates), and base quality score recalibration and local realignment around indels followed the recommended workflow of the GATK toolkit (RealignerTargetCreator, IndelRealigner, BaseRecalibrator, and PrintReads).

#### Targeted sequencing

DNA of eight melanoma samples was isolated and subjected to targeted sequencing of designed ‘Cancer mini-genome’ consisting of 1,977 cancer genes, based on [36]. Pools of libraries were enriched for this gene set using SureSelect technology (Agilent Technologies, Santa Clara, CA, USA). Enriched libraries were sequenced to an average coverage of 150× on a SOLiD 5,500 × l instrument according to the manufacturer’s protocol. Mapping, variant calling, and annotation was done as previously described [36,37].

#### arrayCGH

Mouse SCLC samples were analyzed with Nimblegen 135 K arrayCGH (12 × 135 k WG-T array, 091016_MM9_RK_CGH_HX12) containing 137,221 *in situ* synthesized oligonucleotides (Roche Nimblegen, Madison, WI, USA). Labeling was performed with 250 ng of input DNA according to the manufacturer’s instructions. Image acquisition of the Nimblegen arrays was performed with the Agilent DNA Microarray Scanner (Model G2505B, Serial number US22502518) and image analysis was performed using Nimblescan software version 2.6 (Roche Nimblegen).

#### SNP6 data and pre-processing

Melanoma derived from PDX were run on Affymetrix SNP6 chips for copy number analysis. All arrays were run according to the manufacturers’ instructions. DNA processing, preparation, hybridization, and chip scanning were performed at the Wellcome Trust Sanger Institute (WTSI). The data were normalized using the CRLMM [48] package and HapMap reference data as provided by Affymetrix [49].

#### Whole-genome low-coverage sequencing

Mouse mammary tumors were analyzed by WGS with an average genome coverage of 0.2×, and the same read-count based method was used as applied in CopywriteR. Total read counts for each sample are documented in Additional file 1.

### CopywriteR workflow

Removal of the enriched regions and retrieval of the off-target DOC is performed in multiple steps: (1) reads are filtered for Phred score >37 and for reads mapping in proper pairs (FLAG 0 × 2) using SAMtools; (2) genomic regions enriched for sequencing reads (peaks) are identified in reference samples using MACS 1.4 [50]; and (3) reads corresponding to peak regions identified in step (2) are discarded in sample and reference using bedtools 2.19.1.

DOC ratios for each bin are calculated as follows: (1) DOC is calculated for genome-wide 20 kb bins (unless indicated otherwise) using Rsamtools 1.14.3; (2) DOC is compensated for reduced effective bin sizes upon removal of peak region reads (that is, if x is the cumulative length of all MACS-peaks in base pairs within one 20 kb bin, compensated DOC = uncompensated DOC * 20,000/(20,000 - x)); (3) DOC is corrected for GC content and mappability using two loess normalization steps, and regions of copy number variation are discarded according to the uniqueness of the ENCODE reference genome [34] (these correction steps are implemented in CopywriteR); (4) median normalization and log_2_ transformation of the corrected and compensated DOC; (5) subtraction of log_2_ transformed, corrected, and compensated DOC of the reference sample (for creating relative copy number profiles); and (6) reporting in log files.

All the algorithms and methods have been implemented in the CopywriteR package, which uses R and Unix command-line utilities. CopywriteR takes BAM files from targeted sequencing as input. CopywriteR is parallelized where possible to allow simultaneous processing of multiple samples, and is executed using three functions. The first function generates mappability and GC-content files for the provided bin size. The second function calculates compensated read counts, performs the mappability and GC-content-based normalization steps and applies a filter for regions of germline copy number variation. The results are provided in tab-separated format. The third function is optional and allows segmentation using CBS [29] as implemented in the R-package CGHcall 2.22.0 [42], and plotting of the results. One parallel run of CopywriteR on a desktop computer with a 2.7 GHz CPU and 12 GB of RAM on two samples of 156 and 144 million sequence reads takes under 2 h. Our package (v1.3), as deployed on the data described here, is available for download from GitHub [25].

### Comparison array and SNP methods to WES copy number data

CopywriteR-derived copy number ratios were compared to Affymetrix SNP6 (PDX-derived melanoma) and Nimblegen 135 K (mSCLC) by constructing pseudo counts matching bins of the CopywriteR analysis. More specifically, for creating Nimblegen pseudo counts, the count of a particular bin was set to the intensity of the arrayCGH probe that is nearest to the center of that bin. For Affymetrix SNP6 data, the average intensity of all probes that fall within each bin was set as a pseudo count. Segmentation using CBS [29] was performed on the Nimblegen and Affymetrix pseudo counts with identical settings as for the CopywriteR-derived copy number profiles.

### Use of onTarget, EXCAVATOR, and segmentation methods

The onTarget method is near-identical to ExomeCNV [12] and calculates the mean depth of coverage per base pair for sequence reads that map to a particular capture region for a specific capture set. It follows the CopywriteR workflow, with the difference that it disregards background reads, and that it does not apply peak removal in compensation steps. EXCAVATOR, CBS, and propSeg were used according to the package manuals.

### Distance and correlation calculation

For the calculation of the distance and correlation measures of a set of segmentation values, we first extracted all overlapping genomic regions between the set, and calculated the weighted Euclidian distance and the weighted Pearson product-moment correlation coefficient for these regions (the weighing is based on the length of a specific overlapping region). When applying this to a set of samples, we calculated mean weighted variants of the distance and correlation measures.

### Repositories

Locations of pseudogenes (versions human68 and mouse76) were obtained from pseudogene.org; locations of Ensembl genes (version Ensembl Genes 75) were obtained from the Biomart repository.

## Additional files

Additional file 1:
**Tumor and reference samples used in this study.** Description of the material used for sequencing, and specifications of WES/LC-WGS, depth of coverages, and peaks called by MACS. Additional file 2: Figures S1 to S9. This document contains supplementary **Figures S1-9.**

## Abbreviations

Bp: Base pairs
CBS: Circular binary segmentation
CGH: Comparative genomic hybridization
ChIPseq: Chromatin immunoPrecipitation sequencing
CNA: Copy number aberration
DOC: Depth of coverage
FFPE: Formalin-Fixed Paraffin-Embedded
Indel: Insertion/deletion
Kb: kilobases
MACS: Model-based analysis of ChIPseq
SNP: Single nucleotide polymorphism
WES: Whole-exome sequencing
WGS: Whole-genome sequencing
