# Supplementary material for: CopywriteR: DNA copy number detection from off-target sequence data
Source: Genome Biol. 2015 Feb 27;16(1):49. doi: 10.1186/s13059-015-0617-1 (PMC4396974; doi:10.1186/s13059-015-0617-1)
Supplement: Additional file 2: Figures S1 to S9. — This document contains supplementary Figures S1-9. [file 13059_2015_617_MOESM2_ESM.pdf]

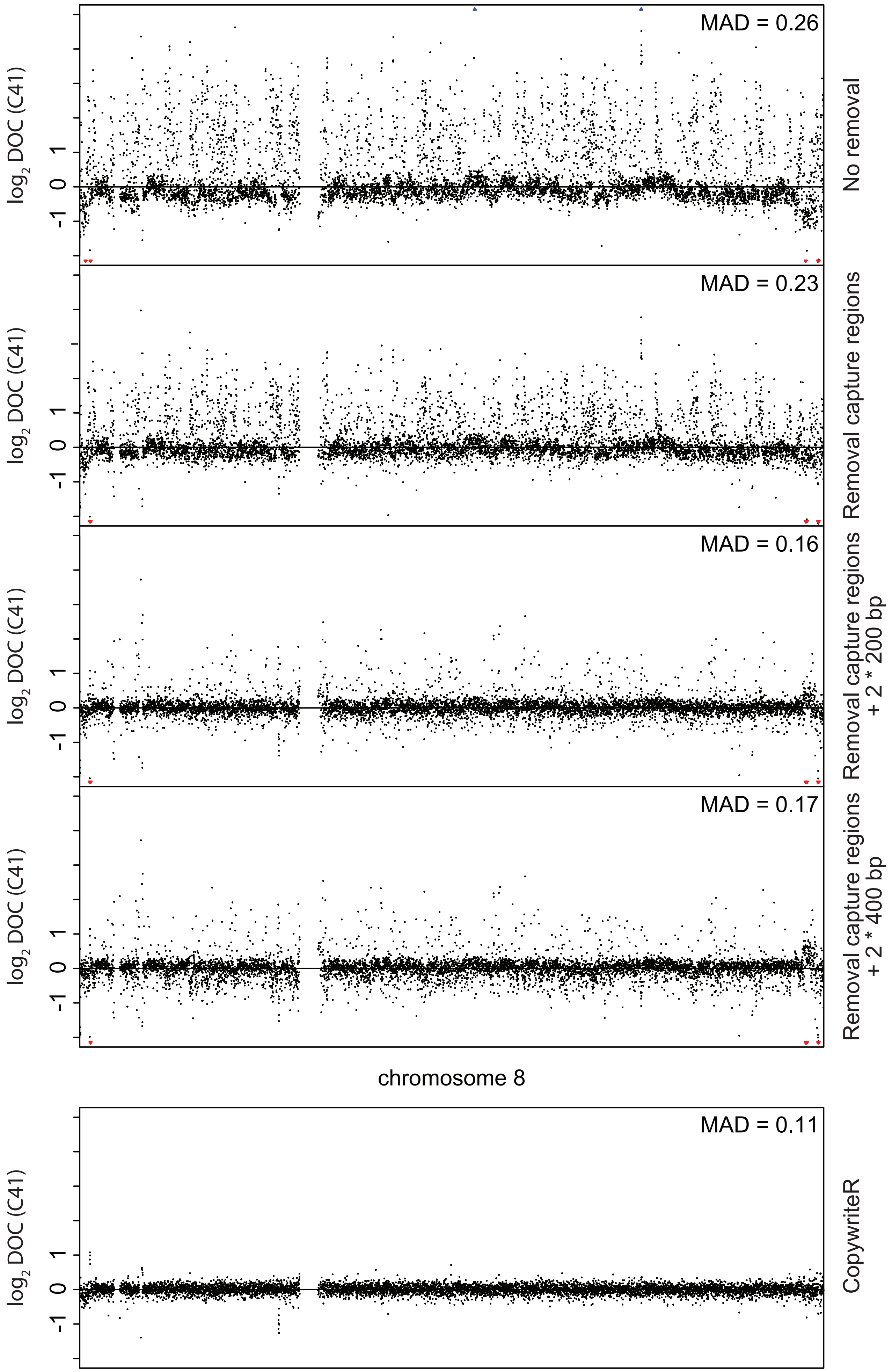

Supplementary Figure S1 Copy number profiles after removal of peaks on capture regions.
Single-channel values of log_2-transformed normalized read count from C41 are plotted. Capture regions were extended with 0, 200 or 400 bp, and sequence reads mapping to these regions were disregarded (middle 3 panels). A copy number profile without removal of any sequence reads is shown in the top panel. The copy number profile after application of the CopywriteR algorithm is displayed at the bottom for comparison.

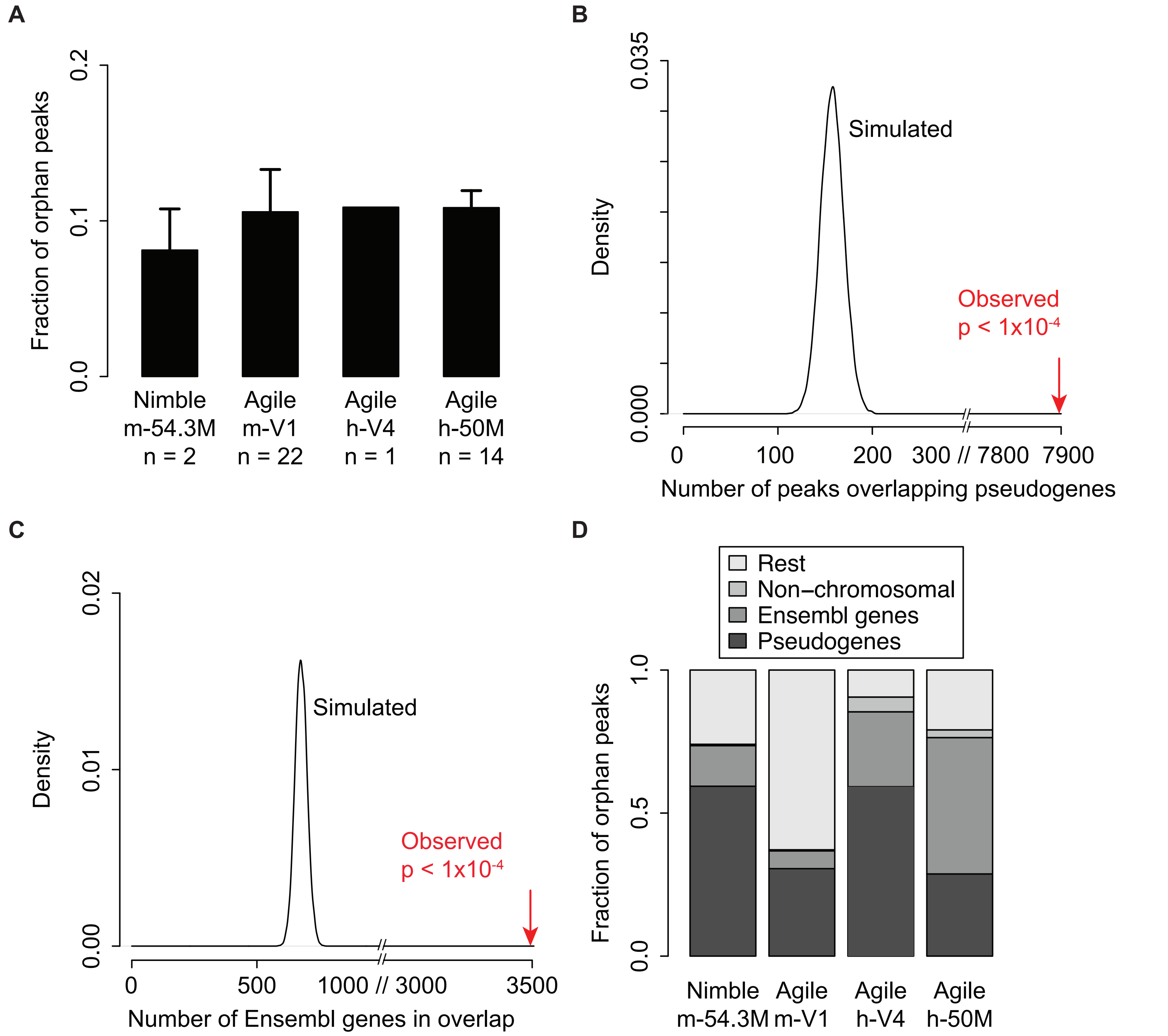

E

	Pseudogenes			Ensembl genes		
	**Simulated**	**Observed**	**Ratio**	**Simulated**	**Observed**	**Ratio**
**Nimble m-53.4M**	75−154	5181	47.1	273-414	1239	3.7
**Agile m-V1**	252−382	9636	30.2	1591-1902	1959	1.1
**Agile h-V4**	114-211	7898	50.3	516-705	3493	5.8
**Agile h-50M**	103-194	5383	37.4	1018-1295	8934	7.6

Supplementary Figure S2 Nature of orphan peaks.
(a) Germline DNA samples were subjected to WES with capture set Agilent SureSelect Mouse all Exon Kit V1. Peaks were identified using MACS and the fraction of those that do not overlap any capture baits are represented. **(b)** Germline DNA sample C41 was subjected to WES with capture set Agilent SureSelect Human Exon Kit V4. Randomly distributed regions were simulated 10,000x with an equal size distribution to the orphan peaks, and the overlap with pseudogenes was calculated. The density distribution of the number of peaks that overlap with pseudogenes for the simulations is shown. **(c)** As in **(b)**, but now the overlap with Ensembl exons was simulated and compared to the actual observed value. **(d)** Germline DNA samples C1, C39, C41 and C45 were subjected to WES with several capture sets. The nature of MACS-peaks that do not overlap capture regions is displayed. The fraction of these orphan peaks that overlap with pseudogene and Ensembl exons, that are non-chromosomal or unmappable, and that do not belong to any of these categories are shown. **(e)** The range of overlap of pseudogenes or Ensembl genes with simulated orphan peaks as in **(d)**, the actual observed overlap, and the ratio of the observed overlap and the median simulated overlap (‘ratio’) are shown for the indicated capture sets.

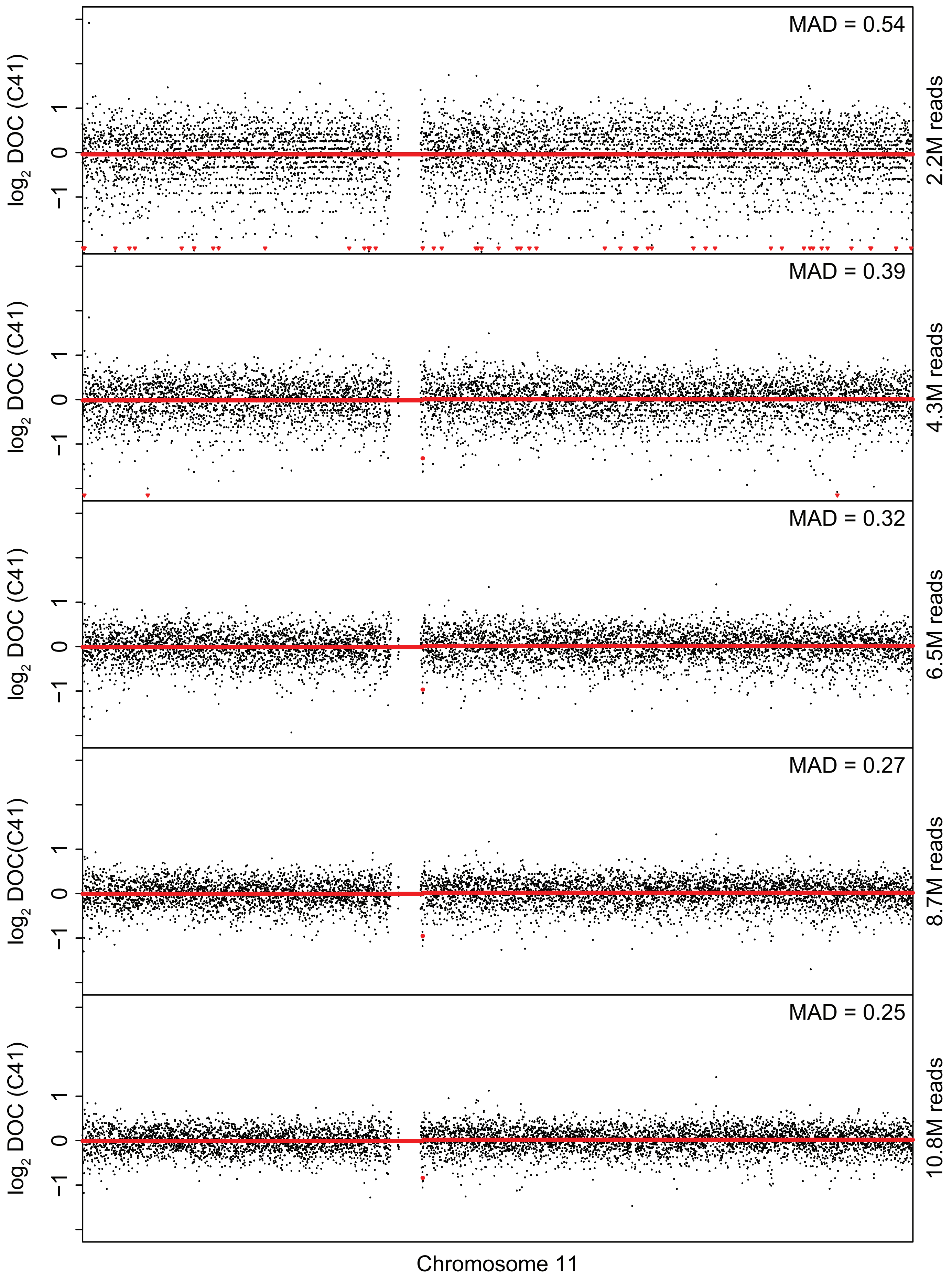

Supplementary Figure S3 The effect of the number sequence read counts on the quality of the copy number profile. Germline DNA sample C41 was subjected to WES, and increasing numbers of random sequence reads are sampled using samtools. Copy number profiles, with segmentation values (CBS) depicted in red, are shown together with MAD-values.

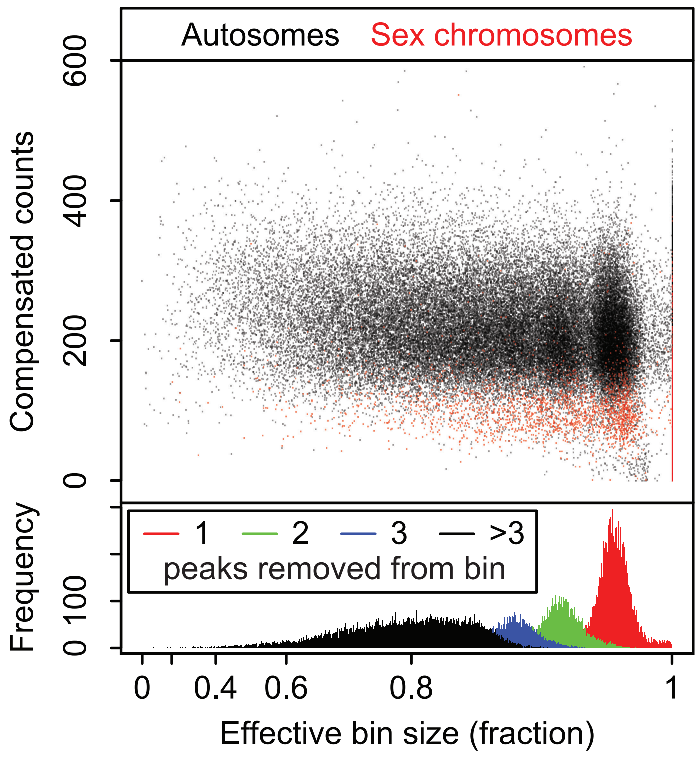

Supplementary Figure S4 Distribution of compensated read counts as a function of the effective bin size. Germline DNA sample C45 was subjected to WES, and the amount of reads after compensation for reduced effective bin size is plotted as a function of the effective bin size upon peak removal (represented as a fraction of the original bin size; upper panel). In the lower panel, a histogram is shown of the compensated reads, subdivided by the number of peaks that are removed from a particular bin.

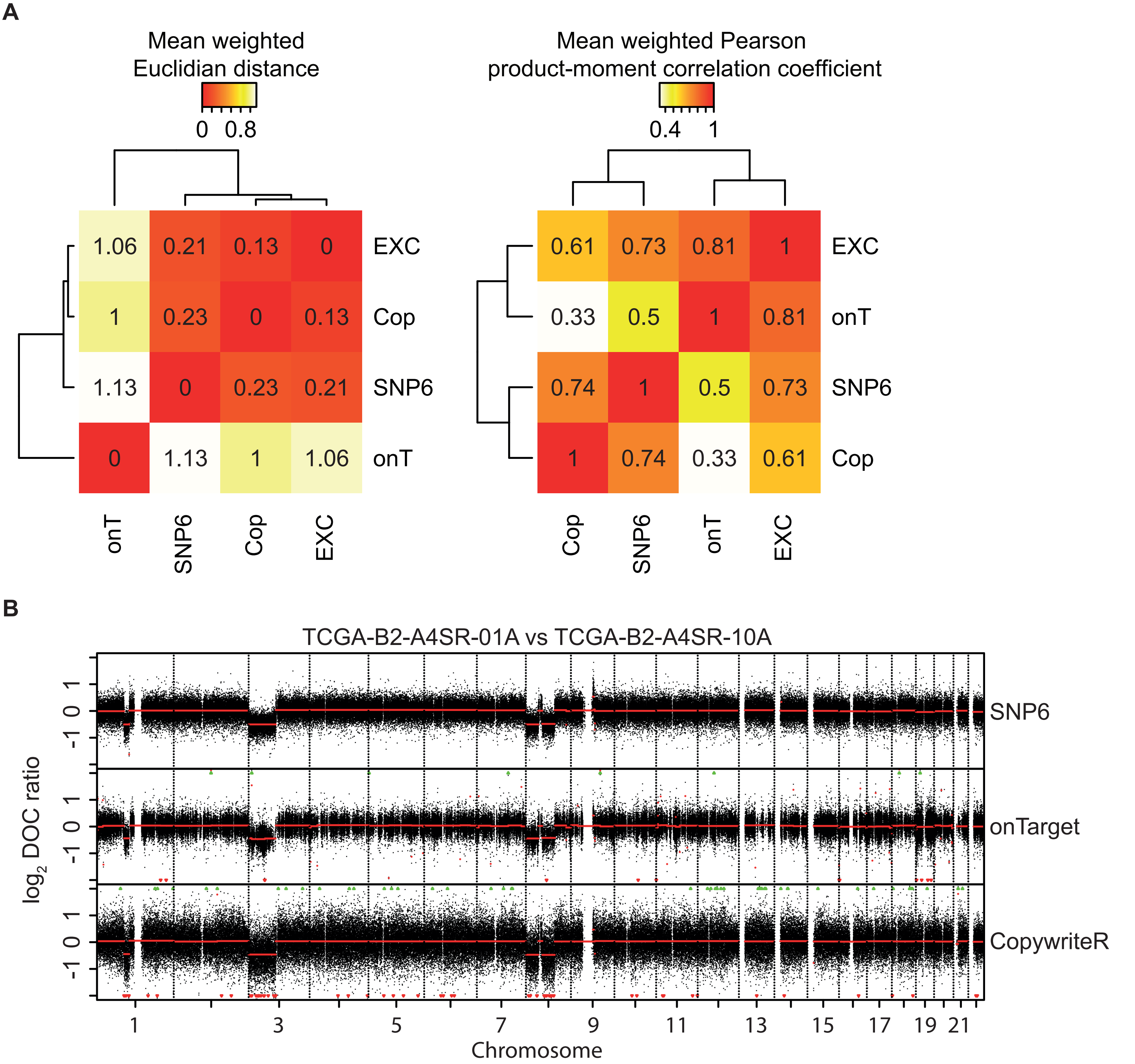

Supplementary Figure S5 The performance of CopywriteR compared to onTarget on an independent sequencing data set. **(a)** Kidney renal clear cell carcinoma sequence data from TCGA were analyzed using CopywriteR and onTarget methods and subsequently segmented using CBS. For comparison, analysis using EXCAVATOR is included, and SNP6-derived copy number data serve as a standard to assess the performance of these methods. The mean weighted Euclidian distance and Pearson correlation coefficient were calculated between segmentation values of all methods, and clustering of this analysis is represented. **(b)** Genome-wide copy number profiles of the analysis in, with segmentation values depicted in red **(a)**.

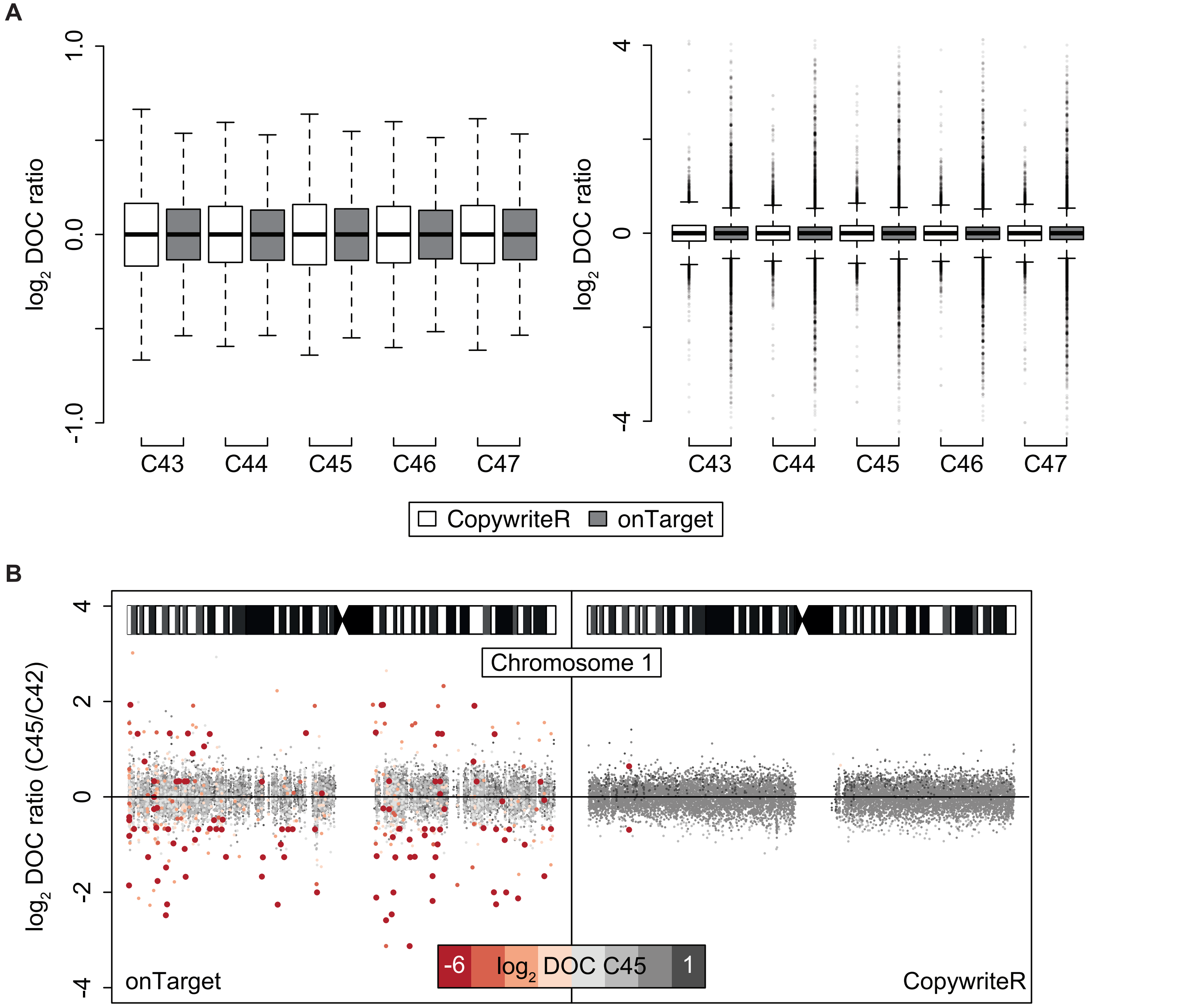

Supplementary Figure S6 Comparison of onTarget and CopywriteR methods.
(a) Boxplots of the corrected compensated depth of coverage of onTarget and CopywriteR methods for the indicated germline DNA samples are shown in the left panel, while the same boxplots including outliers are shown in the right panel. Outliers are displayed with low opacity to visualize the density of data points. **(b)** CopywriteR and onTarget methods were applied to WES data of germline DNA sample C45. Copy number profiles relative to sample C42 are shown for chromosome 1. Data points are colored according to the log2-transformed normalized read count in sample C45.

A

Number of segments per method

	T98	T99	T100	T101	T102	T103
EXCAVATOR	79	133	86	141	137	92
CopywriteR CBS	76	144	192	75	107	143
onTarget CBS	103	188	262	342	570	148
CopywriteR propSeg	95	246	817	75	123	174
onTarget propSeg	830	1534	2316	2154	2389	1087

B

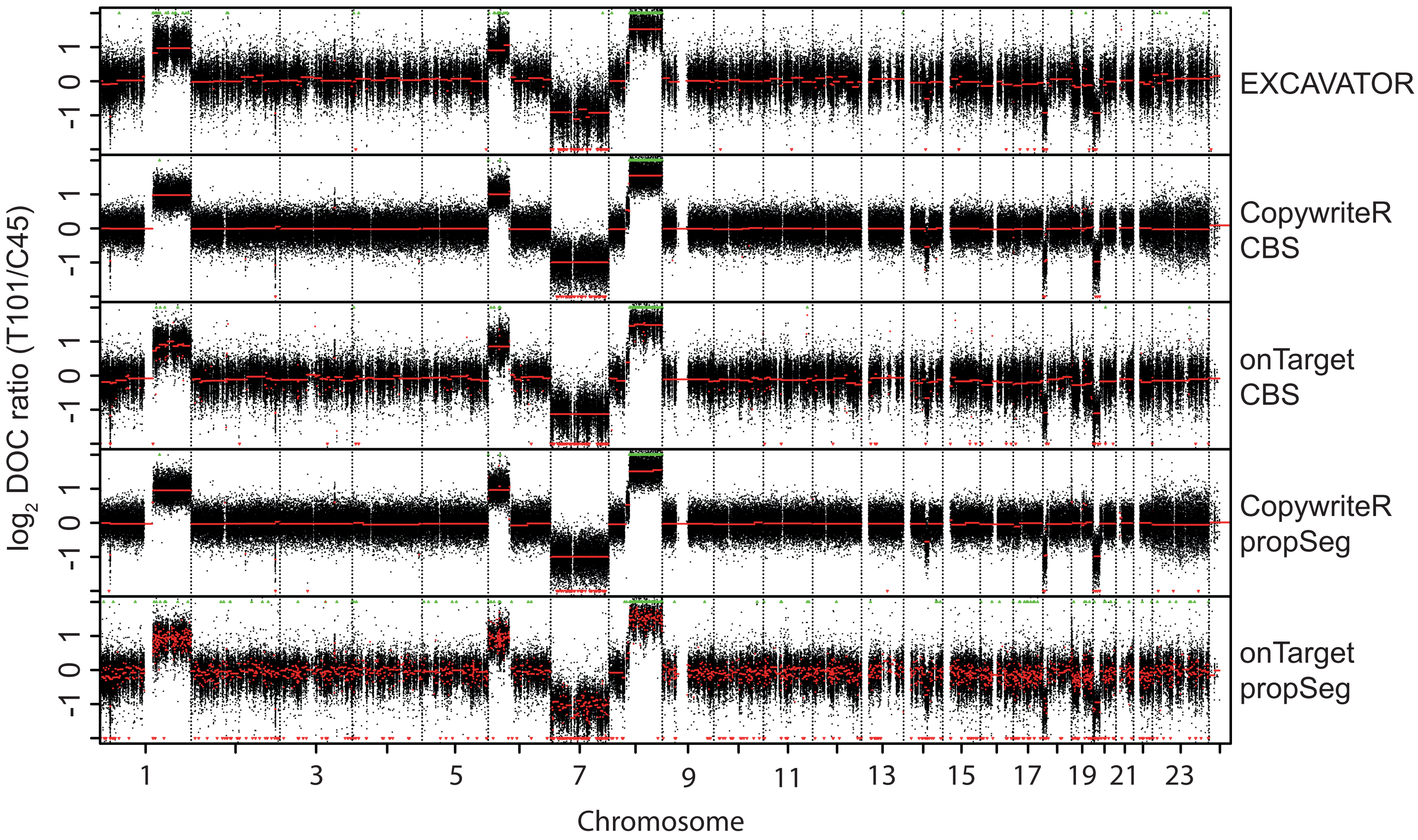

Supplementary Figure S7 Extent of over-segmentation for CopywriteR, onTarget, EXCAVATOR approaches. **(a)** The indicated melanoma PDX samples were subjected to WES, and the amount of segments called with propSeg and CBS algorithms is counted on CopywriteR and onTarget derived data. As a comparison, analysis using EXCAVATOR is included. **(b)** Genome-wide copy number profiles of the analysis in **(a)**, with segmentation values depicted in red.

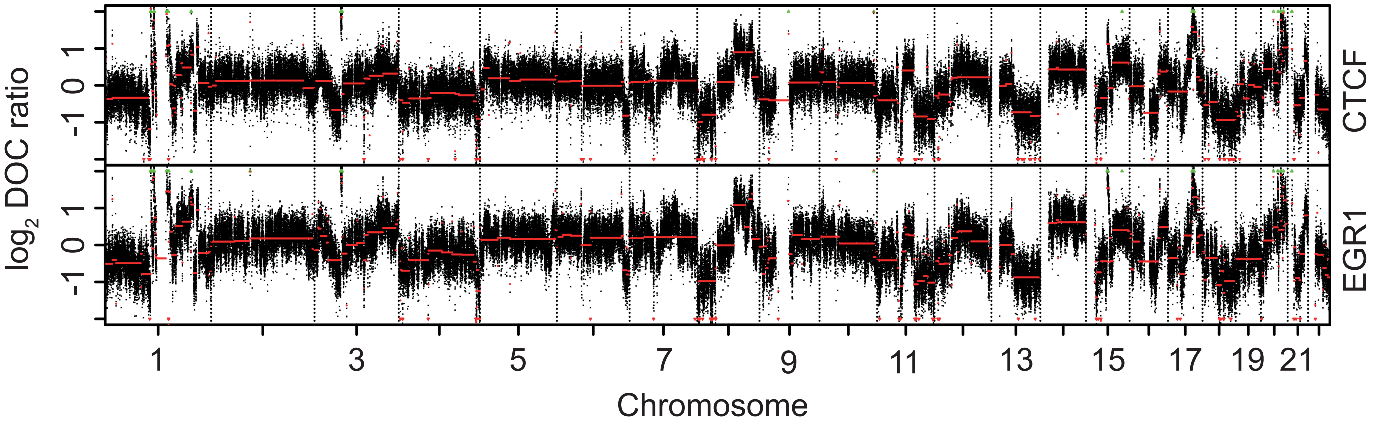

Supplementary Figure S8 Genomide-wide copy number profiles derived from ChIPseq.
ChIPseq data from immunoprecipitations using the indicated antibodies on MCF7 cells was analyzed as described in Figure 5B, and genome-wide copy number profiles, with segmentation values (CBS) depicted in red, are represented.

A

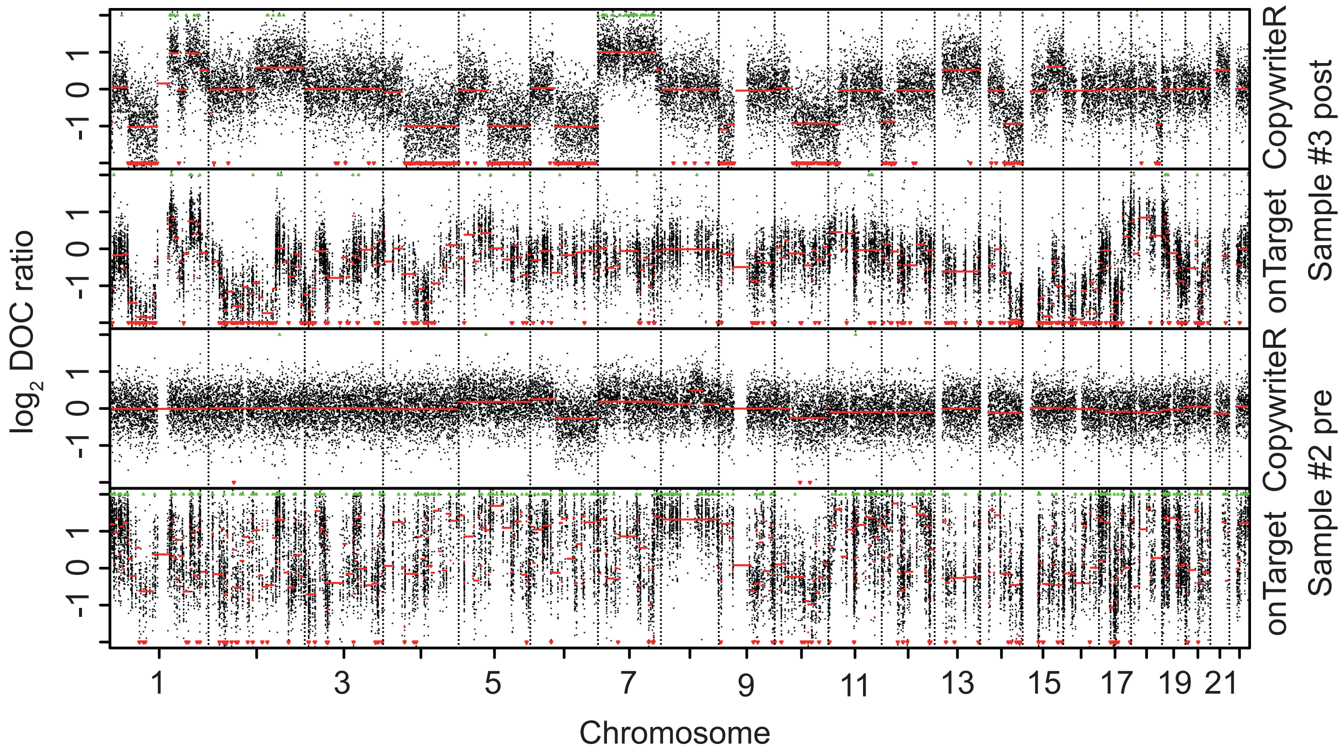

Supplementary Figure S9 Genomide-wide copy number profiles derived from targeted sequencing on a small-size gene panel.
Copy number information of targeted sequencing on a small-size gene panel was extracted as described in Figure 5C, and genome-wide copy number profiles, with segmentation values (CBS) depicted in red, are represented.
